# Neuroprotective Effect of Danhong Injection on Cerebral Ischemia-Reperfusion Injury in Rats by Activation of the PI3K-Akt Pathway

**DOI:** 10.3389/fphar.2020.00298

**Published:** 2020-03-11

**Authors:** Chen Feng, Haofang Wan, Yangyang Zhang, Li Yu, Chongyu Shao, Yu He, Haitong Wan, Weifeng Jin

**Affiliations:** ^1^The Second Clinical Medical College, Zhejiang Chinese Medical University, Hangzhou, China; ^2^Academy of Chinese Medical Sciences, Zhejiang Chinese Medical University, Hangzhou, China; ^3^College of Life Science, Zhejiang Chinese Medical University, Hangzhou, China; ^4^College of Pharmaceutical Sciences, Zhejiang Chinese Medical University, Hangzhou, China

**Keywords:** Danhong injection, neuroprotection, apoptosis, ischemia-reperfusion, PI3K-Akt pathway

## Abstract

Many traditional Chinese medicines, including Danhong injection (DHI), can be used to treat cerebral ischemia-reperfusion injury and have neuroprotective effects on the brain; however, few studies have explored the mechanism by which this effect is generated. In this study, we investigated the neuroprotective effect of DHI against cerebral ischemia-reperfusion injury mediated via the PI3K-Akt signaling pathway. After establishing the model of middle cerebral artery occlusion (MCAO), 60 male Sprague–Dawley rats were allocated to six groups as follows: sham, MCAO, DHI (MCAO + DHI), LY294002 (MCAO + LY294002 [PI3K-Akt pathway specific inhibitor]), DHI + LY294002 (MCAO + DHI + LY294002), and NMDP + LY294002 (MCAO + NMDP [nimodipine] + LY294002). Hematoxylin and eosin (HE) and terminal deoxynucleotidyl transferase dUTP nick-end labeling (TUNEL) staining were used to evaluate the pathological changes of brain tissue and the degree of neuronal apoptosis. Real-time quantitative polymerase chain reaction (qRT-PCR), western blot analysis and enzyme-linked immunosorbent assays were used to measure the expression of Bad, Bax, Bcl-2, Bim, P53, MDM2, Akt, PI3K, p-Akt, p-PI3K, and Cyt-C. Compared with the MCAO group, brain tissue cell apoptosis was significantly reduced in the DHI group, and the brain function score was significantly improved. In addition, the expression of pro-apoptotic factors (Bad, Bax, and Bim) was significantly downregulated in the DHI group, while expression of the anti-apoptotic factor Bcl-2 was significantly upregulated, and expression of the apoptotic gene p53 was also significantly attenuated. Moreover, this neuroprotective effect was attenuated by the PI3K-Akt signaling pathway inhibitor (LY294002). Thus, our results confirmed the neuroprotective effects of DHI in rats with ischemia-reperfusion injury and indicate that these effects on the brain are partly generated by activation of the PI3K-Akt signaling pathway.

## Introduction

Of the many types of cerebrovascular diseases, ischemic cerebrovascular disease is the most harmful (Catanese et al., 2017). Ischemic cerebrovascular disease is characterized by high morbidity and mortality (Dong et al., 2016); however, acute ischemic stroke is the main cause of many disabilities related to brain tissue damage in adults (Boers et al., 2013), Furthermore, acute ischemic stroke accounts for 30% of deaths worldwide. Within a few minutes after the onset of ischemic stroke, brain tissue cells begin to undergo necrosis; therefore, early thrombolytic therapy can restore blood flow in necrotic areas and reduce mortality in patients with ischemic stroke (Christophe et al., 2017). When the blood flow is restored, oxygen is returned to the ischemic area of the brain to rescue and re-establish neurons (Sanderson et al., 2013). *In vivo* and *in vitro* studies have shown that the structure of mitochondria changes during brain ischemia, thereby reducing the supply of energy and the occurrence of acidosis (Verdin et al., 2010). In addition, the process of cerebral ischemia is associated with the release of large amounts of oxygen-free radicals combined with calcium overload and inflammatory reactions (Pinton et al., 2008; Raha and Robinson, 2010).

Numerous studies in recent years have shown that apoptosis plays an important role in ischemic brain damage, especially in reperfusion damage (Chen et al., 2011). The mechanism of apoptosis in the brain ischemia is very complex, and its occurrence is regulated by a variety of genes, including the caspase, the Bcl-2, and p53 gene families (Green and Reed, 1998; Martinou and Youle, 2011). These genes are associated with the PI3K-Akt pathway, which is involved in the regulation of various other cellular functions such as cell proliferation, cell differentiation, and glucose transport (Brazil et al., 2004). Studies have also shown that the PI3K-Akt signaling pathway is involved in neuroprotection against cerebral ischemic injury (Janelidze et al., 2001; Noshita et al., 2001).

To date, many drugs have been used to treat cerebral ischemia-reperfusion injury, but these are associated with problems such as a short therapeutic time window (Lee et al., 2018). Traditional Chinese medicine (TCM) has been practiced for thousands of years (Cheung, 2011) and has made a significant difference in the treatment of certain diseases, including cerebrovascular disease. Traditional Chinese herbal medicine is widely used to treat stroke (Bu et al., 2013; Fu et al., 2014). Since its launch in 2002, Danhong Injection (DHI) has been widely used to prevent and treat a variety of cardiovascular diseases, such as blood reperfusion damage, atherosclerosis, acute coronary artery syndrome and hepatic venous blocking disease (Yao et al., 2011). DHI is formulated from two well-known traditional Chinese medicines, *Salvia miltiorrhiza Bunge* (Danshen in Chinese) and *Carthamus tinctorius L.* (Honghua in Chinese). From the perspective of TCM, these compounds are often used in combination to achieve synergistic effects and reduce side-effects in the treatment of cerebrovascular diseases (Wang et al., 2014; Li et al., 2015). According to previous studies in cerebral ischemia model mice, DHI significantly improves the survival rate and improves neurological symptoms and brain tissue damage after cerebral ischemic injury (Yu et al., 2012; Feng et al., 2018). DHI prevents the development of cerebrovascular thrombosis by promoting the growth of nerve cells and endothelial cells, alleviating local ischemia and hypoxia in the brain, and dilating cerebrovascular vessels and increasing vascular elasticity (Man et al., 2006). Thus, DHI has been shown to exhibit unique advantages in the treatment of cardiovascular and cerebrovascular diseases, although the specific mechanism of action remains to be clarified.

In this study, we evaluated the neuroprotective effect of DHI in a model of ischemia-reperfusion injury established in rats and investigate the potential mechanism by analyzing the expression of important genes and proteins in the PI3K-Akt signaling pathway. Our results provide experimental evidence based on modern pharmacology for the treatment of cerebral ischemic diseases and provides a scientific basis for the clinical use of DHI to treat cardiovascular and cerebrovascular ischemic diseases.

## Materials and Methods

### Reagents and Experiment Animals

**FIGURE 1:**
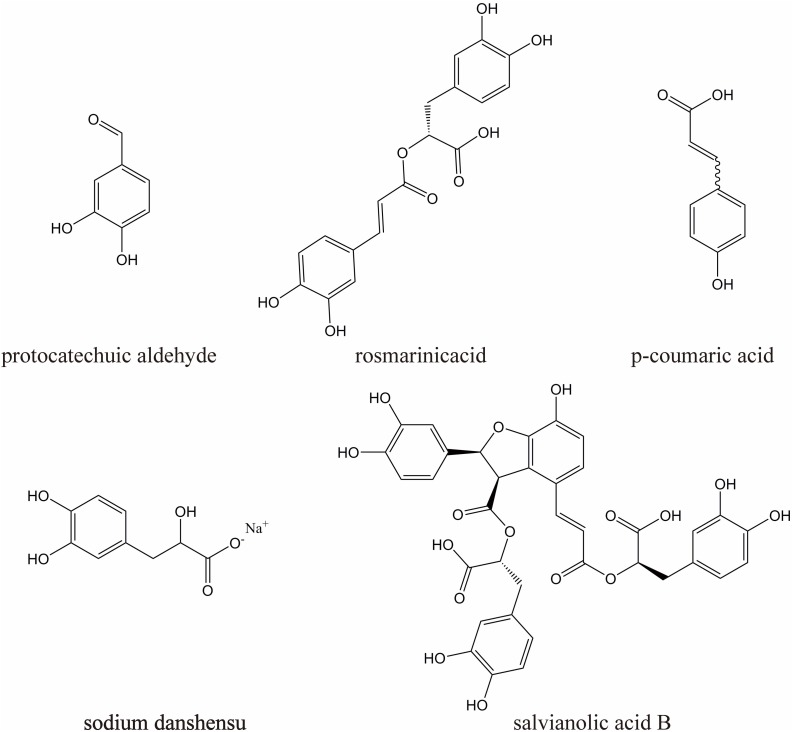
The chemical structures of five major components in DHI.

DHI (10 mL/ampoules, China Food and Drug Administration Permission Number: Z20026866) was provided by Heze Buchang Pharmaceutical Co., Ltd., China. 1000 ml of DHI is prepared from 250 g of *Carthamus tinctorius L*. and 750 g of *Salvia miltiorrhiza Bunge*. China’s State Food and Drug Administration has set clear and strict drug quality control requirements for DHI [The file number is WS-11220(ZD-11220)-2002–2017Z]. According to this standard, the main active substances of DHI are sodium danshensu (C_9_H_9_O_5_Na, not less than 0.80 mg per 1 ml DHI), protocatechuic aldehyde (C_7_H_6_O_3_, not less than 0.10 mg per 1 ml DHI), rosmarinic acid (C_18_H_16_O_8_, not less than 0.10 mg per 1 ml DHI), salvianolic acid B (C_36_H_30_O_16_, not less than 0.16 mg per 1 ml DHI), p-coumaric acid (C_9_H_8_O_3_, not less than 20 ug per 1 ml DHI), their chemical structures are shown in Figure 1.

Hematoxylin stain was purchased from Servicebio Biotechnology (Wuhan, China); Yihong stain was supplied by Besso Biotechnology (Zhuhai, China); TUNEL kit, RIPA lysis and extraction buffer and PMSF were supplied by Biyuntian Biotechnology (Shanghai, China); BCA protein quantification kit, chemiluminescence detection reagent and pre-stained protein markers were purchased from Solarbio Biotechnology (Beijing, China); rat Cytochrome-C (Cyt-C) enzyme-linked immunosorbent assay (ELISA) kit and rat MDM2-P53 binding protein homolog (MDM2) ELISA kit were purchased from Mei Biao Biological Technology (Jiangsu, China); BCA Protein Quantification Kit is supplied by Solarbio Technology (Beijing, China). rabbit anti-mouse monoclonal antibodies against Bad (ab32445, diluted 1:500), Bim (ab32158, diluted 1:500), Bax (ab32503, diluted 1:500), Bcl-2 (ab185002, diluted 1:500), p-Akt (ab38449, diluted 1:500), p-PI3K (ab182651, diluted 1:500), PI3K (ab32089, diluted 1:500), AKT (ab179463, diluted 1:500) and the PI3K-Akt pathway specific inhibitor LY294002 (ab120243) were supplied by Abcam Biotech (Shanghai, China); DHI was purchased from Buchang Pharmaceutical Company (Shandong, China); and Nimodipine was purchased from Dessite Biotechnology (Sichuan, China).

Male Sprague–Dawley rats (aged 6–8 weeks, weighing 260–290 g) were provided by the Animal Experimental Center of Zhejiang Chinese Medicine University (Laboratory animal certificate: scxk 2008-0115) to establish a model of middle cerebral artery occlusion (MCAO). Rats were maintained in a standard feeding cage with free access to food and water. The management and handling of the animals during the trial were in accordance with the International Code of ethics. All procedures were performed in accordance with the Guidelines for the Care and Use of Laboratory Animals (NIH Publications, No. 80-23, revised in 1996). The experiment was approved and supervised by the Institutional Animal Care and Use Committee at Zhejiang Chinese Medical University.

### Middle Cerebral Artery Occlusion (MCAO) Models Establishment

Rats were first anesthetized by intraperitoneal injection of chlorine hydrate (10%, 400 mg/kg). The rat was fixed and then cut along the midline of the neck to reveal the left common carrot artery (CCA), external carrot artery (ECA), and internal carotid artery (ICA). The proximal ends of the ICA, CCA, and ECA were clamped with a micro-arterial clamp. A small portion (4 mm in length) was cut in the CCA and a wire plug was inserted into the ICA. The plug was then inserted gently until a slight resistance was felt. Finally, the distal end of the CCA was ligated and the wound was sutured (Longa et al., 1989; Belayev et al., 1996). A successful model was judged by Horner syndrome in the right eye when the rats awoke up, with its left forelimb bent after lifting the tail, and moved left in a circle as they moved autonomously on the ground. Rats with massive bleeding, subarachnoid hemorrhage, and premature death were excluded after cerebral ischemia-reperfusion injury. Finally, sixty male Sprague–Dawley rats were used in the experiment.

### Animal Grouping

Sixty male Sprague–Dawley rats were allocated to six groups (*n* = 10 per group) as follows: sham, MCAO, DHI (MCAO + DHI), LY294002 [MCAO + LY294002 (PI3K-Akt pathway specific inhibitor)], DHI + LY294002 (MCAO + DHI + LY294002), and NMDP + LY294002 (MCAO + NMDP [nimodipine] + LY294002). The MCAO was established as described in section “Middle Cerebral Artery Occlusion (MCAO) Models Establishment”. In the sham group, the artery was not ligated (only the threading process was performed), and the equivalent volume of physiological saline was administered. LY294002 (specific PI3K/Akt signaling pathway inhibitor) was diluted to 0.5 mg/mL in DMSO to prepare a stock solution. And 10 μL of the stock LY294002 was then injected 30 min prior to modeling. Animals were administered DHI at 0.84 mL/kg, which is equivalent to the human dosage of 8 mL. The conversion formula was as follows: The dose of rats (mL/kg) = 6.3 × the dose of human (mL)/60 kg. The dihydropyridine calcium antagonist NMDP (molecular formula: C_21_H_26_N_2_O_7_) was used as a positive control drug in this experiment, and the dosages of NMDP for rats were 10 mL/kg according to a paper (Ai et al., 2016). DHI, DHI + LY294002 and NMDP + LY294002 groups were administered daily via the tail vein for 3 days. Sham and MCAO groups were given the same amount of physiological saline undergoing the same procedures.

### Behavioral Observation

The degree of ischemia-reperfusion injury in rat brain tissue needs to be evaluated after modeling. According to the Zea–Longa neurological deficit scoring criteria, the neurological function of the rats was recorded based on behavioral changes (Ramya et al., 2010). Using this system, higher scores indicate more severe cerebral ischemia-reperfusion injury. The specific scoring criteria were as follows: 0 points, no symptoms of nerve damage; 1 point, the rat cannot fully extend the contralateral forepaw, indicating a minor neurological defect; 2 points, the rat turns to the temporal side, indicating a moderate neurological deficit; 3points, The rats were slumped to the contralateral side at rest, indicating serious neurological impairment; and 4 points, the rats could not be revived and consciousness was lost, indicating a very serious neurological deficit.

### Measurement of Cerebral Infarction

The brain samples were collected at 72 h after cerebral ischemia and frozen at −20°C for 12 min. Then the brain samples were sliced into 2-mm-thick coronal slices and immediately stained with 2% TTC solution at 37°C for 12 min. Infarct volume was calculated the Image J, which expressed as a percentage of the total volume of slices.

### Cyt-C and MDM2 ELISAs

Serum levels of rat Cyt-C and rat MDM2 were determined using commercial ELISA kits according to the manufacturer’s instructions. All samples were analyzed in triplicate and the absorbance (OD value) of each well of the 96-well plate was measured at a wavelength of 450 nm. Concentrations of rat Cyt-C and rat MDM2 were then calculated with reference to relevant standards.

### HE Staining

Rats were deeply anesthetized with 10% chloral hydrate (300 mg/kg) and fixed on a surgical board placed on a dissection table. The thoracic cavity was exposed and the heart was freed, the perfusion needle was inserted from the left ventricle until reaching the aortic level and fixed, and then frozen sterile saline (4°C) was perfused. The rats were then decapitated and the brain tissue was removed by reperfusion of frozen 4% paraformaldehyde (4°C). Immediately after sacrifice, rat brain tissue was removed and partially dewaxed with xylene (5 mm rat brain tissue). After dewaxed, the samples were washed (5×) using a graded ethanol series (100%, 95%, 80%, and 75% diluted with distilled water). After washing, the tissue was stained with hematoxylin (2 g/L) for 5 min and then rinsed again with distilled water. The sample was then immersed in hydrochloric acid/ethanol (1 ml of concentrated hydrochloric acid mixed with 99 ml of 70% alcohol) for 30 s and then in distilled water for 15 min. Samples were then immersed in eosin solution (1%) for 2 min before rinsing with distilled water. Finally, dehydration was carried out with absolute ethanol, and the tissue was sealed with a neutral resin. Images of the sample were collected by microscopic (NIKON ECLIPSE TI-SR and NIKON DS-U3) photographing (200 × and 400 × microscopic observation).

### TUNEL Staining

Apoptosis-positive cells in the rat brain group were detected using TUNEL staining kits according to the manufacturer’s instructions. Paraffin-embedded sections of rat brain tissue were deparaffinized and washed (3 × for 5 min) with phosphate buffered saline (PBS). Then, 2% proteinase K was added and digested for 30 min before washing (3 × for 5 min) with PBS. The TUNEL mixture was then added, and sections were incubated at 37°C for 30 min before washing (3 × for 5 min) with PBS. Sections were then incubated with POD peroxidase labeling reagent for 30 min at 37°C before washing (3 × for 5 min) with PBS. Thereafter, freshly prepared DAB solution was added. observed under a microscope for 2–6 min, rinsed with water, counterstained, and mounted. After TUNEL staining, sections were observed under an optical microscope. The number of TUNEL-positive (apoptotic) cells in three fields of non-overlapping brain tissue were counted for analysis.

### Western Blotting

Brain tissue samples (100 mg) were placed in Petri dishes containing 1 mL of pre-cooled Lysis Buffer, and homogenized. The homogenate was centrifuged at 12,000 × *g* rpm for 5 min at 4°C. The supernatant was transferred to a pre-cooled centrifuge tube and protein denaturation was carried out by the addition of loading buffer (containing β-mercaptoethanol at a ratio of 50:3) at a sample: loading buffer ratio of 1:4). Samples were boiled for 5 min at room temperature. The total protein concentration of the sample was determined using a BCA protein concentration assay kit. Proteins were then separated by SDS-PAGE concentrated glue and transferred to a PVDF membrane. After washing (3 × for 10 min) with Tris buffered saline-tween (TBST), the PVDF membrane blocked in 5% skimmed milk powder for 2 h. After washing (3 × for 10 min) with TBST, the membrane was incubated (with shaking) overnight at 4°C and with primary detection antibodies (rabbit anti-mouse monoclonal antibodies of Bim, Bax, Bcl-2, p-Akt, p-PI3K, PI3K, and AKT; dilution factors shown in section “Reagents and Experiment Animals “). The next day, membranes were shaken at room temperature for 30 min and then washed (3 × for 10 min) with TBST. Membranes were then incubated with the secondary detection antibody diluted in blocking solution and shaken for 1 to 2 h at room temperature. Subsequently, membranes were washed (3 × for 10 min) with TBST and protein bands were detected using a chemiluminescent reagent (A and B mixed 1:1). Each experiment was repeated three times using the same procedure to obtain an average value.

### qRT-PCR Assay

Total RNA was isolated from rat brain tissue by incubation with TRIzol reagent (1000 μl TRIzol per 200 mg of rat tissue) for 5–10 min. After centrifugation at 12,000 × *g* for 10 min, the supernatant (1.5 mL) was mixed with 200 μL chloroform in a centrifuge tube and centrifuged at 12,000 × *g* rpm for 10 min at 4°C. The supernatant was then mixed with 600 μL of isopropyl alcohol in a new 1.5 mL centrifuge tube and centrifuged at 12,000 × *g* rpm for 10 min at 4°C. After discarding the supernatant, the precipitate was rinsed with 1 mL of 75% absolute ethanol (750 μL absolute ethanol and 250 μL of DEPC water) and then, 1 mL of absolute ethanol. After centrifugation at 12,000 × *g* rpm for 5 min at 4°C, the supernatant was discarded and the RNA was resuspended in 40 μL of DEPC water for storage at −80°C prior to analysis. cDNA was generated by reverse transcription at 42°C for 15 min and 85°C for 5 min. In preparation for qRT-PCR analysis, the samples were thoroughly mixed by vortexing and briefly centrifuged at 4000 × *g* rpm. The reaction system was prepared with 10 μL of UltraSYBR Mixture, 1 μL of PCR Forward Primer (10 μM), 1 μL of PCR Reverse Primer (10 μM), 2 μL of cDNA template and 6 μL of ddH_2_O. The qRT-PCR conditions were as follows: 95°C for 10 min denaturation, followed by 40 cycles of 95°C for 15 s, and 60°C for 60 s. The primer sequences used for qRT-PCR are shown in Table 1.

**TABLE 1:** Primer sequences required for qRT-PCR.

Gene	Forward primer	Reverse primer
Rat p53	ACAGTTAGGGGGTACCTGGC	GCTGTGGTGGGCAGAATATCAT
Rat GADPH	GCGGGAGCGGATCCTAATA	TGGTGCATCCATGGGCTAC

### Statistical Analysis

Data analysis was performed using SPSS 24.0 statistical software (SPSS Inc., Chicago, IL, United States). All data were expressed as mean ± standard deviation ($\bar{x}$ ± s), and *P* < 0.05 was be used as the criterion for statistical significance. In pairwise comparisons between groups, *t*-tests were used for two independent samples with homogeneity of variance, and Kruskal–Wallis *H* tests were used for those with heterogeneity of variance.

## Results

### Nerve Function Assessment

**FIGURE 2:**
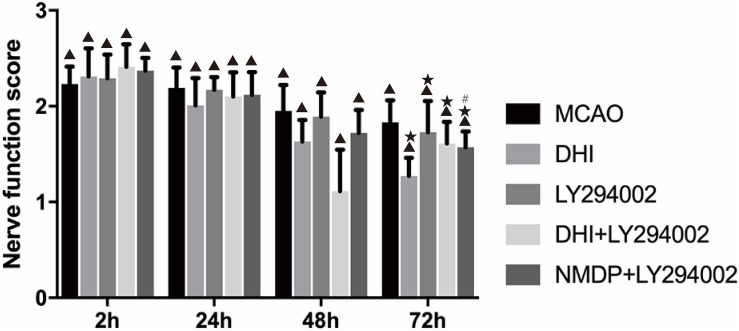
The neurological scores of rats in each group at 2, 24, 48, and 72 h after cerebral ischemia-reperfusion injury (*n* = 10). Compared with the Sham group, ^▲^*p* < 0.05; compared with the MCAO group, *p < 0.05; compared with the LY294002 group, ^#^*p* < 0.05. Sham, sham operation group; MCAO, middle cerebral artery occlusion; LY294002, 2-(4-morpholinyl)-8-phenyl-chromone; DHI, Danhong Injection; NMDP, nimodipine.

Neurological function scores of each group were shown in Figure 2. No prominent changes were observed in the scores (all 0 points) of the sham group at 2, 24, 48, and 72 h after brain ischemia-reperfusion injury. Different degrees of nerve function damage were observed in the other five groups, with all scores significantly higher than those in the sham group (*P* < 0.05). Excluding the sham group, the scores in the other groups decreased over time, reaching their lowest at 72 h. In comparison with the MCAO group, the scores in the DHI, LY294002, DHI + LY294002, and NMDP + LY294002 groups were significantly lower at 72 h (*P* < 0.05). Furthermore, the score in the NMDP + LY294002 group was significantly lower than that in the LY294002 group at 72 h (*P* < 0.05), while there was no significant difference compared with the score in the DHI + LY294002 group (*P* > 0.05).

### Cerebral Infarct Volume Assessment

**FIGURE 3:**
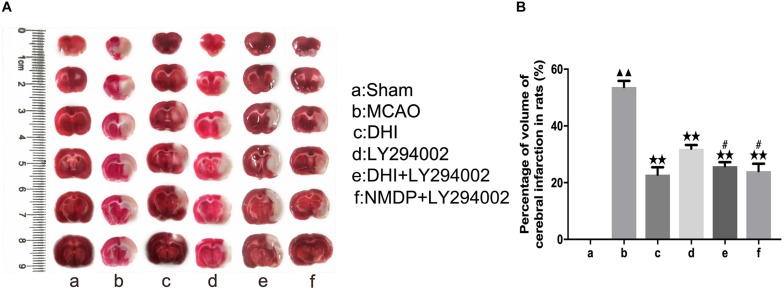
The cerebral infarct volume of rats after cerebral ischemia-reperfusion injury by MCAO with TTC staining. **(A)** Results of TTC staining in each group. **(B)** Statistical results of cerebral infarct volume in each group (*n* = 3). Compared with the sham group, ^▲▲^*p* < 0.01; compared with the MCAO group, **p < 0.01; compared with the LY294002 group, ^#^*p* < 0.05. Sham, sham operation group; MCAO, middle cerebral artery occlusion; LY294002, 2-(4-morpholinyl)-8-phenyl-chromone; DHI, Danhong Injection; NMDP, nimodipine.

As shown in Figure 3, TTC (2,3,5-triphenyltetrazolium chloride) staining showed deep red in the viable tissue and white color in the right infarcted hemisphere. There was no significantly cerebral infarction in the sham group. Compared with the sham group, the infarct volume was increased in the MCAO group (*P* < 0.01). Moreover, the infarct volume was significantly decreased in the DHI, LY294002, DHI + LY294002, and NMDP + LY294002 groups in comparison to the MCAO group (*P* < 0.01). Compared with the LY294002 group, the infarct volume was decreased in the DHI + LY294002 and NMDP + LY294002 groups (*P* < 0.05).

### Histopathological Changes in the Hippocampus

**FIGURE 4:**
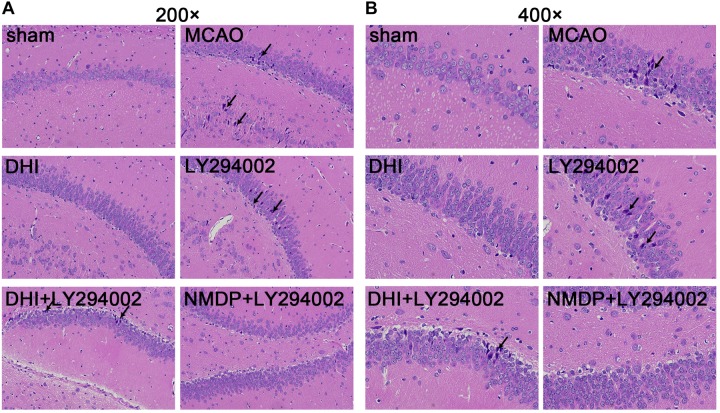
Histopathological changes in hippocampus tissues of rats in each group were observed by HE staining. **(A)** The hippocampus tissues of each group rats at 200-fold magnification after HE staining, and necrotic cells indicated by black tips. **(B)** The hippocampus tissues of each group rats at 200-fold magnification after HE staining, and necrotic cells indicated by black tips (*n* = 3). Sham, sham operation group; MCAO, middle cerebral artery occlusion; LY294002, 2-(4-morpholinyl)-8-phenyl-chromone; DHI, Danhong Injection; NMDP, nimodipine.

As shown in Figure 4, hippocampal neurons and glial cells in the sham group were neatly arranged, with normal structures. The hippocampal nerve cells in the MCAO and LY294002 groups were disorganized, with disrupted cell membrane and swollen cell morphology, loss and death of a large number of neurons, and partial nuclear dissolution and condensation. In comparison to the MCAO group, neuronal and glial cell necrosis, nuclear condensation, cell membrane and cell structure destruction were improved in the DHI group. Compared with the LY294002 group, neuronal necrosis was reduced in the DHI + LY294002 and NMDP + LY294002 groups, with obvious pathological improvement.

### Degree of Apoptosis in Brain Tissue Cells

**FIGURE 5:**
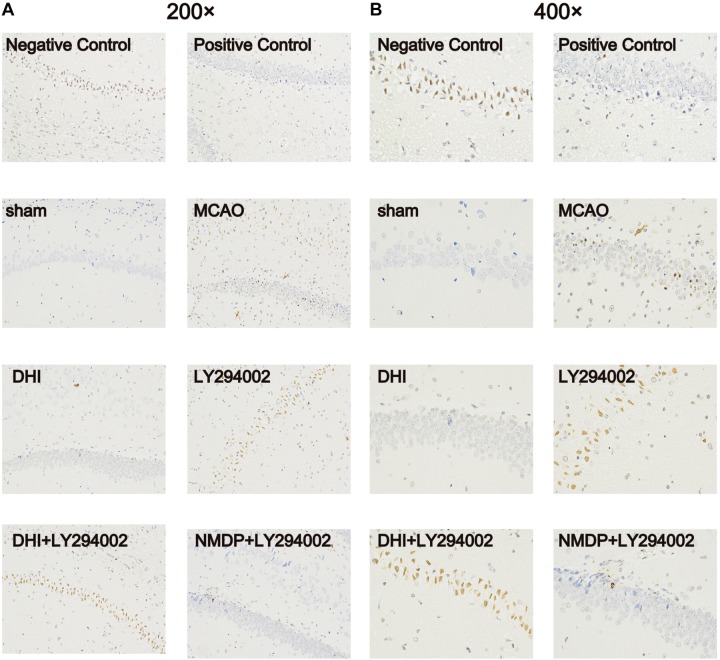
Apoptosis of brain tissue of rats in each group after TUNEL staining. **(A)** The apoptosis of rat brain tissue after TUNEL staining was observed under 200-fold magnification. **(B)** The apoptosis of rat brain tissue after TUNEL staining was observed under 400-fold magnification (*n* = 3). Sham, sham operation group; MCAO, middle cerebral artery occlusion; LY294002, 2-(4-morpholinyl)-8-phenyl-chromone; DHI, Danhong Injection; NMDP, nimodipine.

As shown in Figure 5, no apoptotic cells (brown-yellow stained) were found in the brain tissue in the sham group. A large number of apoptotic cells were observed in the hippocampus of the MCAO and LY294002 groups. In comparison to the MCAO group, fewer apoptotic of neurons were observed in the DHI group. Furthermore, neuronal apoptosis in the DHI + LY294002 and NMDP + LY294002 groups was reduced compared to that in the LY294002 group. These findings indicated that DHI has an anti-apoptotic effect after ischemia-reperfusion injury, and this effect is blocked by the PI3K-Akt pathway inhibitor.

### Serum Levels of Cytochrome-C and MDM2

**FIGURE 6:**
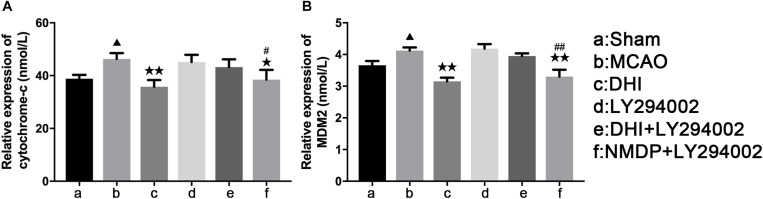
The serum levels of rat Cyt-C and rat MDM2 of each group. **(A)** The expression level of cytochrome-c. **(B)** The expression level of MDM2 (*n* = 10). Compared with the sham operation group, ^▲^*p* < 0.05; compared with the MCAO group, *p < 0.05, **p < 0.01; compared with the LY294002 group, ^#^*p* < 0.05, ^##^*p* < 0.01. Sham, sham operation group; MCAO, middle cerebral artery occlusion; LY294002, 2-(4-morpholinyl)-8-phenyl-chromone; DHI, Danhong Injection; NMDP, nimodipine.

As shown in Figure 6, serum levels of Cyt-C and MDM2 of rats in the MCAO group were significantly increased compared with those in the sham group (*P* < 0.05). In comparison to the MCAO group, Cyt-C expression levels were significantly decreased in the DHI (*P* < 0.01) and NMDP + LY294002 groups (*P* < 0.05), and MDM2 expression levels were also significantly decreased in both groups (*P* < 0.01). In contrast, there was no significant differences in Cyt-C and MDM2 expression levels between the LY294002 and DHI + LY294002 groups (*P* > 0.05). In comparison with the LY294002 group, significantly decreased expression levels of Cyt-C (*P* < 0.05) and MDM2 (*P* < 0.0) were detected in the NMDP + LY294002 group, while there was no significant differences compared with the other groups (*P* > 0.05). These results indicated that DHI inhibits apoptosis and neuronal activity by reducing the level of Cyt-C and inhibiting MDM2 expression in brain tissue.

### The Protein Expressions of PI3K-Akt Pathway Related Proteins in Each Group

**FIGURE 7:**
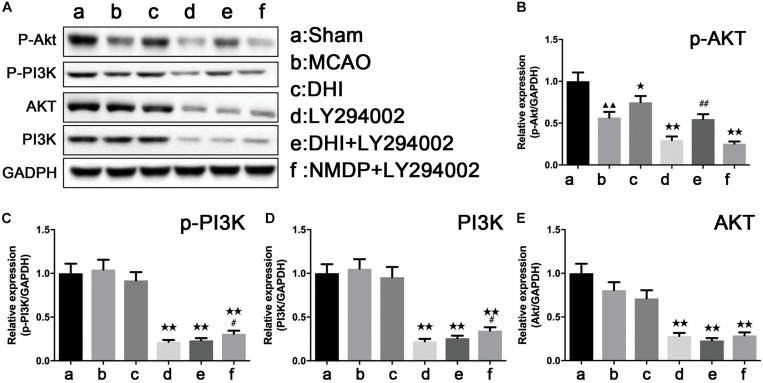
The protein strip diagram and protein expressions level of PI3K-Akt pathway related proteins in each group. **(A)** Band diagrams of p-Akt, p-PI3K, Akt, and PI3K in brain tissue of each group of rats determined by western blot. **(B)** The expression of p-Akt in brain tissue of each group rats determined by western blot. **(C)** The expression of p-PI3K in brain tissue of each group rats determined by western blot. **(D)** The expression of Akt in brain tissue of each group rats determined by western blot. **(E)** The expression of PI3K in brain tissue of each group rats determined by western blot (*n* = 4). Compared with the sham operation group, ^▲▲^*p* < 0.01; compared with the MCAO group, *p < 0.05, **p < 0.01; compared with the LY294002 group, ^#^*p* < 0.05, ^##^*p* < 0.01. Sham, sham operation group; MCAO, middle cerebral artery occlusion; LY294002, 2-(4-morpholinyl)-8-phenyl-chromone; DHI, Danhong Injection; NMDP, nimodipine.

As shown in Figure 7, p-Akt protein expression was significantly downregulated in the MCAO group compared with that in the sham group (*P* < 0.01), while there were no significant differences in the expression of p-PI3K, PI3K, and Akt proteins (*P* > 0.05). Furthermore, p-Akt protein expression in the DHI group was significantly higher than that in the MCAO group (*P* < 0.05). Compared with the DHI group, a significant decrease of the expression levels of p-PI3K, PI3K, and Akt proteins were observed in brain tissues of the DHI + LY294002 group (*P* < 0.01). The expression levels of p-Akt, p-PI3K, PI3K, and Akt proteins were all significantly decreased in brain tissues of the NMDP + LY294002 group compared with the MCAO group (*P* < 0.01). The expression of p-Akt protein in brain tissue in the DHI + LY294002 group was significantly upregulated in comparison to that in the LY294002 group (*P* < 0.01), while there were no significant differences compared with the other groups (*P* > 0.05). The expression of p-PI3K and PI3K proteins in the brain tissues of the NMDP + LY294002 group were significantly enhanced compared with the LY294002 group (*P* < 0.05), while no significant changes were observed in the other groups (*P* > 0.05). These findings indicated that DHI protects against brain ischemia-reperfusion injury by activating the PI3K-Akt signaling pathway in a rat model.

### Expression of Apoptosis-Related Factors

**FIGURE 8:**
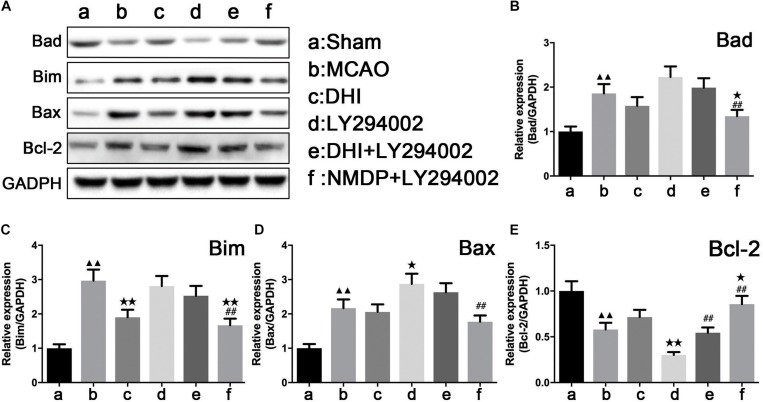
The protein strip diagram and protein expressions level of apoptosis-related factors in each group. **(A)** Band diagrams of Bad, Bim, Bax, and Bcl-2 in brain tissue of each group of rats determined by western blot. **(B)** The expression of Bad in brain tissue of each group rats determined by western blot. **(C)** The expression of Bim in brain tissue of each group rats determined by western blot. **(D)** The expression of Bax in brain tissue of each group rats determined by western blot. **(E)** The expression of Bcl-2 in brain tissue of each group rats determined by western blot (*n* = 4). Compared with the sham operation group, ^▲▲^*p* < 0.01; compared with the MCAO group, *p < 0.05, **p < 0.01; compared with the LY294002 group, ^##^*p* < 0.01. Sham, sham operation group; MCAO, middle cerebral artery occlusion; LY294002, 2-(4-morpholinyl)-8-phenyl-chromone; DHI, Danhong Injection; NMDP, nimodipine.

As shown in Figure 8, expression levels of Bad, Bim, and Bax proteins in the brain tissue in the MCAO group were significantly upregulated compared with those in the sham group (*P* < 0.01), while Bcl-2 protein expression levels were significantly downregulated (*P* < 0.01). In comparison to the MCAO group, only Bim protein expression in the brain of the DHI group was significantly decreased (*P* < 0.01). A significant decrease of Bax and Bcl-2 protein expression in the brain tissues of the LY294002 group was observed (*P* < 0.05), while Bcl-2 protein expression was significantly decreased (*P* < 0.01). In the brain tissues of the NMDP + LY294002 group, Bcl-2 protein expression levels were significantly upregulated (*P* < 0.05), while the expression levels of Bad and Bim proteins were significantly downregulated (*P* < 0.01). Bcl-2 protein expression in brain tissue of the DHI + LY294002 group was significantly increased in comparison with the LY294002 group (*P* < 0.01), while no significant changes were observed in the other groups (*P* > 0.05). A significant decrease in Bad, Bim, and Bax protein expression was observed in the brain tissues of the NMDP + LY294002 group (*P* < 0.01), whereas a significant increase in Bcl-2 protein expression was observed (*P* < 0.01). These findings suggested that the expression of anti-apoptosis-related factors was promoted by DHI, while the expression of pro-apoptotic-related factors was inhibited. Furthermore, the anti-apoptotic effect of DHI was inhibited by LY294002.

### p53 mRNA Expression

**FIGURE 9:**
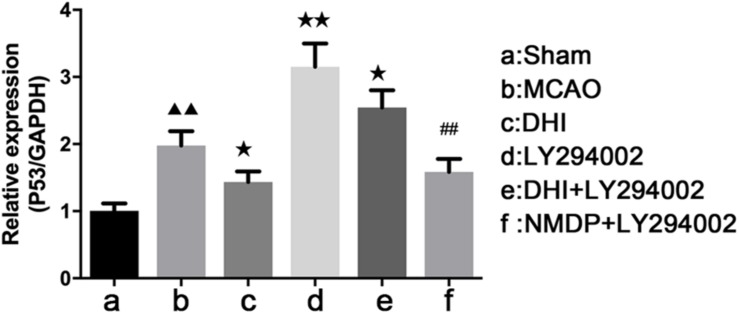
The p53 mRNA expression level in each group determined by qRT-PCR (*n* = 4). Compared with the sham operation group, ^▲▲^*p* < 0.01; compared with the MCAO group, *p < 0.05, **p < 0.01; compared with the LY294002 group, ^##^*p* < 0.01. Sham, sham operation group; MCAO, middle cerebral artery occlusion; LY294002, 2-(4-morpholinyl)-8-phenyl-chromone; DHI, Danhong Injection; NMDP, nimodipine.

As shown in Table 2 and Figure 9, the expression of p53 mRNA in the brain tissues of the MCAO group was significantly increased compared with that in the sham group (*P* < 0.01). In comparison with the MCAO group, decreased p53 expression was detected in the DHI (*P* < 0.05) and NMDP + LY294002 groups (*P* > 0.05). Expression of p53 in LY294002 group and DHI + LY294002 groups was increased significantly (*P* < 0.01 or *P* < 0.05). Compared with the LY294002 group, the expression of p53 mRNA was significantly decreased in both the DHI + LY294002 group (*P* < 0.05) and the NMDP + LY294002 group (*P* < 0.01). These results suggested that DHI inhibited apoptosis by downregulation of p53 gene expression, and LY294002 attenuated this effect.

**TABLE 2:** Expression of p53 mRNA in rat brain tissue ($\bar{x}$ ± s, *n* = 3).

Group	p53 mRNA expression
Sham	1.00 ± 0.11
MCAO	1.98 ± 0.21^▲▲^
DHI	1.43 ± 0.16*
LY294002	3.15 ± 0.35**
DHI + LY294002	2.55 ± 0.25*
NMDP + LY294002	1.58 ± 0.19^##^

Compared with the sham operation group, ^▲▲^P < 0.01; compared with the MCAO group, *P < 0.05, **P < 0.01; compared with the LY294002 group, ^##^P < 0.01. I/R, ischemia-reperfusion; MCAO, middle cerebral artery occlusion; LY294002, 2-(4-morpholinyl)-8-phenyl-chromone; DHI, Danhong Injection; NMDP, nimodipine.

## Discussion

Brain tissue is very sensitive to nerve damage and apoptosis as a result of ischemia-reperfusion injury (Douzinas et al., 2001). In this study, we confirmed the neuroprotective effect of DHI on brain ischemia-reperfusion damage and provided evidence that this effect is mediated via the PI3K-Akt pathway, since inhibiting the PI3K-Akt signaling pathway weaken the neuroprotective effect of DHI on brain ischemic reperfusion damage in a rat MCAO model.

Ischemia-reperfusion damage is caused by many factors including the production of oxygen free radicals (Watson and Ginsberg, 2010), which play a crucial role in neuronal apoptosis. Free radicals also alter the response of blood vessels by damaging endothelial cells and disrupt the blood-brain barrier. Nitric oxide is also involved in acute brain injury and induce apoptosis of nerve cells (Liu et al., 2006). In addition, studies have shown that excitatory amino acids (EAA), which is toxic to brain cells, play an important role of ischemia (Choi et al., 2014). Studies have also shown that the presence of energy metabolism disorders in the brain ischemia leads to a lack of ATP in cells and a decrease in pH, which seriously inhibits the activity of the Na^+^/Ca^2+–^exchange protein, causing an ion imbalance in the cells, which eventually leads to the destruction of the cellular defense system in the brain (Liu et al., 1991). The local inflammatory reactions associated with brain ischemia reperfusion are a major cause of reperfusion damage (Mu et al., 2011), although apoptosis is the main cause of cell death under these conditions (Lipton, 1999). Apoptosis is a gene-regulated active death process, in which caspase-3, Bcl-2, and Bax genes play important regulatory roles.

Activation of Akt plays an important role in neuronal survival following cerebral ischemia-reperfusion injury. Studies have shown that Akt1 overexpression reduces the infarct size after cerebral ischemia by 35% in MCAO model rats (Ohba et al., 2004). Recent studies have also shown that Aktl has an important protective effect on ischemia-reperfusion injury in rats, with Akt overexpression shown to reduce the volume of infarcted brain tissue by 50% (Giuseppe et al., 2008). Therefore, the activation of Akt-mediated neuroprotection has been confirmed in a large number of studies of many agents, such as GDNF, VEGF and erythropoietin, which mediate their neuroprotective effects via the PI3K-Akt signaling pathway (Jin et al., 2003; Dilaver et al., 2005; Kilic et al., 2005). In accordance with these studies, our study provides evidence that the neuroprotective effects of DHI are also mediated via the PI3K-Akt pathway.

Both the p53 and MDM2 genes are associated with apoptosis (Polyak et al., 1997; Ray et al., 2011), and are also important in signaling downstream of Akt (Seger, 2002). These genes have been shown to play important roles in neuronal apoptosis, senescence and cell cycle arrest. It has been reported that p53 negatively regulates the PI3K-Akt pathway; therefore, the Akt-MDM2-p53 axis forms a negative feedback loop to regulate p53 expression and block p53-mediated pro-apoptotic gene transcription (Kim et al., 2001). In our study, we found that p53 and MDM2 gene expression was significantly increased in the MCAO group compared with that in the sham group, while the levels were significantly reduced in the DHI group. When the activation of PI3K-Atk pathway was inhibited, the effects of DHI were greatly attenuated, indicating that the mechanism underlying the neuroprotective effects of DHI involves regulation of p53 gene expression via PI3K-Atk signaling pathway.

Mitochondria are organelles that play a key regulatory role in neuronal apoptosis signaling pathways (Green and Reed, 1998). Phosphorylation of PI3K-Akt leads to upregulation of mitochondrial transcription factors and cytochrome c oxidase, resulting in decreased ATP production and cell death (Zhou et al., 2000). Cyt-C is a water-soluble protein encoded by a nuclear gene located outside the mitochondrial body. Cyt-C functions as an electron carrier in the mitochondrial respiratory chain, which plays an important role in the mitochondrial energy metabolism (Song et al., 2017). During apoptosis, Cyt-C is released into the cytoplasm, where it binds to apoptosis activation factor 1 (Apaf-1), and spontaneously activates caspase-9 to form a Cyt-C/Apaf-1/caspase-9 complex (Jiang et al., 2017). This further activates the caspase family, causing cell necrosis and DNA fragmentation which results in apoptosis (Jiang and Wang, 2000). Studies have shown that in the early stages of apoptosis, Cyt-C is released from the mitochondrial membrane to initiate the process of cellular apoptosis. Furthermore, Cyt-C release is induced by expression of the pro-apoptosis gene Bax, the anti-apoptosis gene Bcl-2 blocks the release of Cyt-C and activation of caspases (Jürgensmeier et al., 1998). Both Bax and Bcl-2 are downstream proteins in the PI3K-Akt signaling pathway that regulate the release of Cyt-C in mitochondria and the activation of caspase.

The components of TCM are very complicated and can contain dozens of compounds. There are also many precious compounds in the extracts of Rhizoma *Salviae Miltiorrhizae* and Flos Carthami contain many other important compounds including Tanshinone, Salvianic acid A, Hydroxysafflor Yellow A, and Safflower Yellow B, which have significant effects in the treatment or prevention of cardiovascular and cerebrovascular diseases.

Sodium danshensu is one of the important ingredients in Danshen and one of the quality control standards of DHI. Shao et al. found that sodium danshensu has a neuroprotective effect on the brain of rats with cerebral ischemia-reperfusion injury, and the related mechanism may be by activating the PI3K/Akt pathway to inhibit apoptosis (Guo et al., 2015). Zhang et al. (2017) discovered through research that rosmarinic acid protects rat hippocampal neurons from cerebral ischemia-reperfusion injury through the Akt/JNK3/caspase-3 pathway. And rosmarinic acid is also an important component in DHI, and its content is also one of the quality inspection standards of DHI. There are also two main components of DHI: salvianolic acid B and p-coumaric acid. Fan et al. found that salvianolic acid B has neuroprotective effect on brain injury induced by ischemia-reperfusion injury in rats by reducing the generation of free radicals, and which may be an effective clinical candidate treatment (Fan et al., 2018). During the research, Sakamula et al. found that pretreatment with p-coumaric acid can significantly reduce malondialdehyde levels, whole cerebral infarct volume, and hippocampal neuron death, and increase catalase and superoxide dismutase activities, eventually producing neuroprotective effect (Sakamula and Thong-asa, 2018).

Xu et al. showed that Tanshinone IIA exerts a significant cardioprotective effect by improving heart function and reducing the infarct size (Wei et al., 2009). Liu et al. found that Salvianic acid A inhibited cerebrovascular endothelial apoptosis induced in mice by hydrogen peroxide via the PI3K/Akt/Raf/MEK/ERK pathway (Chen-Li et al., 2007). Zhong et al. demonstrated that Salvianic acid A reduced the number of apoptotic nerve cells after ischemia in rats through the restoration of movement (Jing et al., 2007). Lin et al. demonstrated that Hydroxysafflor Yellow A can prevent brain ischemic reperfusion damage By reducing the expression of genes involved in brain cell apoptosis via the PI3K/Akt/GSK3 beta signaling pathway (Lin et al., 2013). In a study of the protective effect of Danshensu and Hydroxysafflor Yellow on ischemic reperfusion injury in mice, Xu et al. showed that the drug combination had a better protective effect on nerve cells in mice than the individual drugs alone (Xu et al., 2017). Du et al. found that Safflower Yellow B protected the brain ischemic reperfusion damage by inhibiting AMPK-mediated NF-κB activation and reducing the expression of inflammatory cytokines (Du et al., 2019). The evidence provided in this study confirm the reliable neuroprotection against ischemia-reperfusion injury provided by DHI, which is a combination of two TCM active components.

It has been reported that The Jak2-STAT3 signaling pathway also plays an important role in the protection of the brain against ischemia-reperfusion damage (Irawan et al., 2010) via a mechanism that involves the PI3K-Akt signaling pathway (Hou et al., 2018). Further studies are required to explore the relationship between the neuroprotective effect of DHI on brain ischemia-reperfusion injury and Jak2-STAT3 signaling pathways, as well as the interaction effect between the Jak2-STAT3 and PI3K-Akt signaling pathways.

## Conclusion

In summary, our results demonstrate that DHI can protect brain tissue from ischemia-reperfusion injury in rats by reducing the inflammatory response and apoptosis of brain tissue cells. And we found that DHI produces this neuroprotective effect by regulating the expression of important proteins and genes in the PI3K-Akt pathway, indicating that this signaling pathway may be the mechanism behind this protective effect. The findings of this study provide a reference for the clinical anti-apoptosis and neuroprotection of DHI.

## Data Availability Statement

The datasets generated for this study are available on request to the corresponding author.

## Ethics Statement

The animal study was reviewed and approved by the Institutional Animal Care and Use Committee at Zhejiang Chinese Medical University.

## Author Contributions

WJ and HW conceived and designed the study. CF, HW, and YZ were tested and analyzed the data. CF and HW wrote this manuscript. LY, YH, and CS coordinated the research and provided the technical assistance. WJ, HW, and LY modified the file. All authors reviewed the results and approved the final version of the manuscript.

## Conflict of Interest

The authors declare that the research was conducted in the absence of any commercial or financial relationships that could be construed as a potential conflict of interest.
